# A novel integrated nutrition-combined prognostic index for predicting overall survival after radical gastrectomy

**DOI:** 10.3389/fnut.2024.1438319

**Published:** 2024-11-18

**Authors:** Xiang Li, Zhongxue Fu, Jun Zhang, Jinming Xu, Lianwei Wang, Ke Li

**Affiliations:** ^1^Department of Gastrointestinal Surgery, Chongqing University FuLing Hospital, Chongqing, China; ^2^Department of General Surgery, The Third Affiliated Hospital of Chongqing Medical University, Chongqing, China; ^3^Department of Gastrointestinal Surgery, The First Affiliated Hospital of Chongqing Medical University, Chongqing, China

**Keywords:** gastric cancer, nutrition-combined prognostic index, nomogram, overall survival, nutrition

## Abstract

**Objective:**

The objectives of this study were to integrate the Prognostic Nutritional Index, Controlling Nutritional Status, and Nutritional Risk Index, into a novel Nutrition-combined Prognostic Index (NCPI), and to develop and validate a nomogram to predict overall survival (OS) in patients with gastric cancer (GC).

**Materials and methods:**

Data from 609 patients with GC, collected between January 1, 2017, and April 30, 2023, were retrospectively analyzed. Optimal cut-off values for nutritional parameters were determined using X-Tile software, and the Kaplan–Meier method applied for survival analysis. Univariate, least absolute shrinkage and selection operator, and multivariate Cox regression analyses were conducted, and a nomogram for predicting OS in patients with GC constructed and validated.

**Results:**

Inferior nutritional status was strongly correlated with worse clinicopathologic features and prognosis of patients with GC. NCPI, body mass index, American Joint Committee on Cancer T stage, and lymph node ratio were identified as independent risk factors for OS. A nomogram including these factors predicted 1-, 3-, and 5-year OS, with training and validation set C-index values of 0.716 and 0.77, respectively. Calibration curves demonstrated that the predicted outcomes closely matched the actual results, and decision curve analysis highlighted the high practical value of the model.

**Conclusion:**

The novel nutritional marker, NCPI, is closely associated with the clinicopathologic features and OS of patients with GC. The practical value of the NCPI-based nomogram was demonstrated and a web-based calculator developed.

## 1 Introduction

Gastric cancer (GC) is among the most prevalent malignant tumors (GLOBOCAN)^1^ (1), and results in high mortality, contributing significantly to the global disease burden (2). Surgery remains the primary treatment for resectable gastric tumors, and offers the sole curative option (3). Adequate surgical resection and meticulous lymph node dissection are crucial to preventing postoperative recurrence. Health status and tolerance of the side effects caused by surgery and systemic therapy significantly influence patient prognosis (4, 5). Hence, nutritional status is critical in patients with GC.

More broadly, clinicians are increasingly recognizing the significance of the nutritional status of oncology patients (6), where nutritional status serves as a potential prognostic factor that can be addressed prior to surgery (7). In cases of severe nutritional risk, the European Society for Clinical Nutrition and Metabolism guidelines (8) advise that administration of preoperative nutritional support is essential, even if it requires a delay to surgery.

There are several clinical tools that can be applied to evaluate patient nutritional status, including: the Simple Nutrition Screening Tool (9), Patient-Generated Subjective Global Assessment (10), Subjective Global Assessment (11), Nutritional Risk Screening 2002 (12), and the Global Leadership Initiative on Malnutrition (13); however, these assessment methods are cumbersome, complex, and time-consuming, making them difficult to apply in routine practice. Additionally, some of the data required to complete these scales are not readily available or are significantly flawed due to subjective processes used in their collection; for example, some patients may struggle to recall and provide accurate nutritional data (14). Hence, according to Xiao et al., clinicians face challenges in accurately assessing patient nutritional status and evaluating the efficacy of nutritional interventions using traditional assessment tools (15), underscoring the need for a simple, practical, and objective method to assess patient nutritional status.

The Prognostic Nutritional Index (PNI) integrates lymphocyte and serum albumin data to reflect the nutritional and immune status of patients with cancer (16), and is associated with the prognosis of patients with breast, colorectal, and non-small cell lung cancers (17, 18). The Controlled Nutritional Status (CONUT) scoring system was recently introduced to evaluate nutritional status, based on serum albumin, total cholesterol levels, and lymphocyte counts (19). Lee et al. emphasized the importance of the CONUT score in assessing the nutritional status of patients with gastrointestinal or lung tumors (20). Further, the Nutritional Risk Index (NRI), which was initially introduced by Buzby in 1988 (21), is used to evaluate nutritional status based on weight and serum albumin levels, and serves as an indicator of malnutrition in hospitalized adults (22). The French Nutrition and Health Program recommends application of the NRI to assess the nutritional status of hospitalized adults (23).

The relationships of PNI, CONUT, and NRI scores with GC prognosis remain contentious, and no study has combined the PNI, COUNT, and NRI systems to predict overall survival (OS) in patients with GC. In this study, we separately assessed the correlations of these indices with OS in patients with GC. Additionally, we introduce a novel nutritional parameter, the Nutrition-Combined Prognostic Index (NCPI), that integrates the PNI, CONUT, and NRI. Finally, we developed and validated a nomogram based on the NCPI for predicting OS in patients with GC.

## 2 Materials and methods

### 2.1 Study subjects, inclusion criteria, and exclusion criteria

Clinicopathological data of patients diagnosed with GC who underwent radical surgical operation between January 1, 2017, and April 30, 2023, at the Department of Gastrointestinal Surgery, the First Hospital Affiliated of Chongqing Medical University, and the Department of Gastrointestinal Surgery, Chongqing University FuLing Hospital, were retrospectively analyzed. The inclusion criteria were: a. postoperative histological validation of gastric carcinoma, b. patients who had received curative operation for gastric carcinoma, and c. age ≥ 18 years. The exclusion criteria were: a. patients with concurrent multi-site *in situ* tumors; b. history of other malignant tumors; c. neoadjuvant therapy prior to surgery; d. systemic immune diseases or other significant comorbidities; e. recent use of hormones, immunosuppressants, or similar medications; and f. incomplete clinicopathological data.

### 2.2 Clinicopathological data

Data on the following variables were collected: age, sex, body mass index (BMI), underlying diseases, tumor size, primary site, American Joint Committee on Cancer (AJCC) TNM stage, histological differentiation, pathological classification, positive lymph nodes, total lymph nodes, lymph node ratio (LNR), chemotherapy, postoperative complications, and OS. BMI was defined as weight (kg)/height(m) (2). Underlying diseases considered were hypertension, diabetes mellitus, coronary atherosclerotic heart disease, chronic obstructive pulmonary disease (stable phase), and cerebral infarction. Disease count was determined as follows: patients were scored 0 if none of the specified diseases were present, 1 for one disease, and 2 for two diseases, etc. AJCC TNM stage was as defined by the AJCC and the Union for International Cancer Control (24). LNR was the number of positive lymph nodes divided by the total number of lymph nodes. Postoperative adjuvant therapy was conducted according to the National Comprehensive Cancer Network guidelines (25). Postoperative complications included fistulas, infections (lung, incision, abdominal, etc.), and other issues (bleeding, bowel obstruction, etc.). OS was measured from the date of surgery to last follow-up, patient loss, death from any cause, or follow-up cutoff.

### 2.3 Nutritional biomarkers

Blood sampling and collection of relevant nutritional markers were completed within 24 h of admission for patients diagnosed with GC and intended for surgery (26). PNI (16) was calculated as follows:


$$PNI=\mathrm{serum}\;\mathrm{albumin}\;\left(g/L\right)+5\times \mathrm{lymphocytes}\;\left(\times 10\;\left(9\right)/L\right)\text{.}$$

CONUT is a newly proposed scoring system that calculates a nutritional assessment score, based on serum albumin, total cholesterol, and lymphocyte counts, and is mainly used to assess the nutritional status of patients (19) (Table 1). NRI (21) was calculated as:


$$\begin{array}{l} NRI=1.519\times \mathrm{albumin}\;\left(g/L\right)+41.7\\ \;\times \left(\mathrm{present weight}/\mathrm{ideal weight}\right)\text{.} \end{array}$$

**Table 1:** Definition of controlling nutritional status (CONUT).

Parameters	Normal	Mild malnutrition	Moderate malnutrition	Severe malnutrition
Albumin(g/L)	≥35	30–34.9	25–29.9	<25
Score	0	2	4	6
Lymphocyte(/mm3)	≥1,600	1,200–1,599	800–1,199	<800
Score	0	1	2	3
Cholesterol(mg/dl)	≥180	140–179	100–139	<100
Score	0	1	2	3
Total score	0–1	2–4	5–8	9–12

where ideal body weight formulae (27) were:


$$\mathrm{Male}:\mathrm{height}\;\left(\mathrm{cm}\right)-100-\left[\left(\mathrm{height}\;\left(\mathrm{cm}\right)-150\right)/4\right]\text{.}$$

$$\mathrm{Female}:\mathrm{height}\;\left(\mathrm{cm}\right)-100-\left[\left(\mathrm{height}\;\left(\mathrm{cm}\right)-150\right)/2.5\right]\text{.}$$

Patients were stratified according to the risk of malnutrition as follows: NRI = 100, no risk; NRI = 97.5–100, mild risk; NRI = 83.5–97.5, moderate risk; NRI ≤ 83.5, significant risk.

### 2.4 Follow-up

Follow-up visits were conducted every 3 months during the initial 2 years post-surgery, followed by visits every 6 months thereafter (25). Data were collected from hospital records and patient or family telephone calls, and encompassed details including missed appointments, last known survival dates, or dates of demise. Overall mortality status encompassed all causes of death post-surgery, ensuring comprehensive tracking of patient outcomes over time.

### 2.5 Survival analysis and establishment of independent risk factors for OS

X-Tile software^2^ was used to determine optimal threshold values for nutritional markers, including PNI, CONUT, and NRI. Data were then stratified into high and low nutritional groups, based on parameter cut-off values. Subsequently, differences in clinicopathological characteristics and OS were compared between two groups. Univariate Cox, least absolute shrinkage and selection operator (LASSO), and multivariate Cox regression analyses were employed to identify independent prognostic factors influencing the prognosis (OS) of patients with GC, using the aforementioned nutritional parameters. Hazard ratio (HR) and 95% confidence interval (CI) values were calculated accordingly.

### 2.6 Establishment and validation of prognostic model for GC

Initially, patient data were randomly divided into training and validation sets (ratio, 7:3). Subsequently, independent prognostic factors identified in the previous step were utilized to construct a nomogram prediction model for OS at 1, 3, and 5 years. C-index values were calculated to compare the model performance with that of traditional TNM staging. Time-area under the curve (AUC) values for 1-, 3-, and 5-year OS were used to evaluate the predictive ability of the model in the training and validation datasets. Calibration curves were used to assess the agreement between predicted and actual OS. Decision curve analysis was applied to compare the model performance in predicting patient OS at 1, 3, and 5 years with that of traditional TNM stage. Patient scores were calculated and categorized into high, medium, and low-risk groups using X-Tile software, to evaluate survival differences. Finally, a dynamic web calculator was developed, based on the prediction model.

### 2.7 Statistical analysis

Statistical analyses were conducted using R software (version 4.3.1).^3^ Categorical variables are expressed as frequencies and percentages and were compared using either the chi-square (χ^2^) or Fisher’s exact probability tests. Continuous variables are described using median and interquartile range. Between-group comparisons of normally distributed continuous variables were made using the independent samples t-test (Student’s test), while the Mann–Whitney rank sum test (Mann–Whitney U test) was applied to compare non-normally distributed continuous variables. Significance was determined at *p* < 0.05 (two-tailed).

## 3 Results

### 3.1 Patient clinicopathologic characteristics

**Figure 1:**
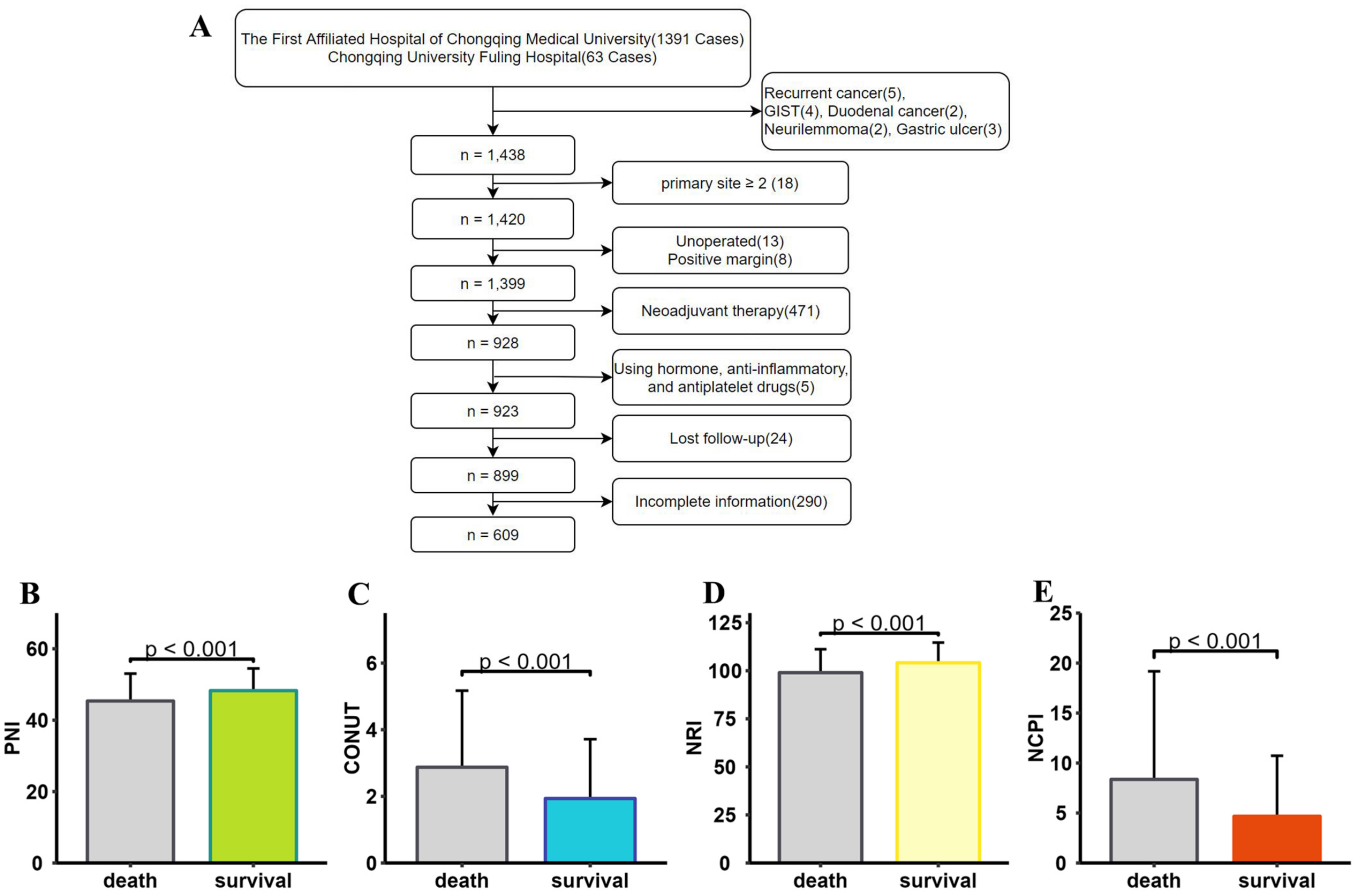
Data screening and histogram demonstrating that patients who died (*N* = 150) and those who survived (*N* = 459) had significantly different nutritional status (*p* < 0.001). **(A)** Flowchart showing the patient screening process. PNI values were significantly lower **(B)**, CONUT scores were significantly higher **(C)**, NRI values were significantly lower **(D)**, and NCPI values were significantly higher **(E)** in deceased patients than those in surviving patients. PNI, Prognostic Nutritional Index; CONUT, Controlling Nutritional Status; NRI, Nutritional Risk Index; NCPI, Nutrition-Combined Prognostic Index.

We conducted a retrospective analysis of medical records from patients diagnosed with GC and admitted for surgical treatment at two affiliated hospitals between January 1, 2017, and April 30, 2023. Initially, 1,391 patients from one hospital and 63 from the other were reviewed, yielding a final dataset of 609 patients after applying our inclusion and exclusion criteria (Figure 1A). Among these, 150 patients had deceased, while 459 were living at the time of analysis. Significant disparities in sex distribution, age, BMI, tumor characteristics, AJCC staging, lymph node involvement, chemotherapy, and postoperative complications were observed between the two groups; patients who had died were of higher median age, with larger tumor size, advanced stage tumors, more positive lymph nodes, and a higher incidence of postoperative complications than survivors (Table 2). Differences in PNI, CONUT, and NRI were also detected between patients who survived and those who died (Figures 1B–D).

**Table 2:** Baseline characteristics of patients with gastric cancer.

Characteristic	Overall *N* = 609	death *N* = 150	survival *N* = 459	*p*-value
Gender (%)				0.03
Female	194 (32%)	37 (25%)	157 (34%)	
Male	415 (68%)	113 (75%)	302 (66%)	
Age	66 (59, 72)	67 (62, 75)	66 (58, 71)	0.003
Underlying diseases (%)				0.14
0	424 (70%)	95 (63%)	329 (72%)	
1	137 (22%)	40 (27%)	97 (21%)	
2	43 (7.1%)	15 (10%)	28 (6.1%)	
3	4 (0.7%)	0 (0%)	4 (0.9%)	
4	1 (0.2%)	0 (0%)	1 (0.2%)	
BMI, median (IQR)	22.76 (20.57, 24.61)	22.04 (19.73, 23.76)	22.86 (20.82, 24.80)	<0.001
Tumor size, median (IQR)	3.00 (1.80, 4.00)	3.50 (2.50, 4.50)	2.50 (1.50, 3.50)	<0.001
Histological differentiation (%)				0.016
Well differentiated	9 (1.5%)	0 (0%)	9 (2.0%)	
Moderately differentiated	139 (23%)	32 (21%)	107 (23%)	
Poorly differentiated	419 (69%)	114 (76%)	305 (66%)	
Unclassified	42 (6.9%)	4 (2.7%)	38 (8.3%)	
Pathological classification (%)				0.003
Adenocarcinoma	497 (82%)	131 (87%)	366 (80%)	
Mucinous carcinoma	10 (1.6%)	4 (2.7%)	6 (1.3%)	
Signet-ring cell carcinoma	70 (11%)	13 (8.7%)	57 (12%)	
Adenosquamous carcinoma	1 (0.2%)	0 (0%)	1 (0.2%)	
Squamous cell carcinoma	5 (0.8%)	2 (1.3%)	3 (0.7%)	
Carcinoma	26 (4.3%)	0 (0%)	26 (5.7%)	
AJCC T_Stage (%)				<0.001
Tis	65 (11%)	1 (0.7%)	64 (14%)	
T1	150 (25%)	9 (6.0%)	141 (31%)	
T2	79 (13%)	20 (13%)	59 (13%)	
T3	7 (1.1%)	3 (2.0%)	4 (0.9%)	
T4	308 (51%)	117 (78%)	191 (42%)	
AJCC N_Stage (%)				<0.001
N0	326 (54%)	42 (28%)	284 (62%)	
N1	95 (16%)	27 (18%)	68 (15%)	
N2	79 (13%)	27 (18%)	52 (11%)	
N3	109 (18%)	54 (36%)	55 (12%)	
LNR, median (IQR)	0.00 (0.00, 0.19)	0.18 (0.00, 0.48)	0.00 (0.00, 0.09)	<0.001
Positive_LN, median (IQR)	0.0 (0.0, 4.0)	4.0 (0.0, 9.8)	0.0 (0.0, 2.0)	<0.001
Total_LN, median (IQR)	22 (17, 28)	21 (16, 27)	22 (17, 28)	0.3
Primary site (%)				0.2
Upper third	121 (20%)	37 (25%)	84 (18%)	
Middle third	281 (46%)	67 (45%)	214 (47%)	
Lower third	207 (34%)	46 (31%)	161 (35%)	
Chemotherapy (%)	370 (61%)	122 (81%)	248 (54%)	<0.001
Complications (%)				0.047
Fistula	47 (7.7%)	14 (9.3%)	33 (7.2%)	
Infection	29 (4.8%)	13 (8.7%)	16 (3.5%)	
Other	26 (4.3%)	5 (3.3%)	21 (4.6%)	
None	507 (83%)	118 (79%)	389 (85%)	

IQR, Interquartile Range; BMI, Body Mass Index; LN, Lymph Nodes; LNR, Lymph Node Ratio.

### 3.2 Nutrition-combined prognostic index

Univariate and multivariate Cox regression analyses were conducted, with univariate analysis revealing associations of OS with PNI, CONUT score, and NRI (Supplementary Table 1); however, multivariate analysis indicated that these factors were not independent risk factors for OS (Supplementary Table 1). Consequently, guided by our univariate findings, we devised a novel parameter, the NCPI, calculated as:


$$\mathrm{NCPI}=\frac{10000\times \mathrm{CONUT}}{\left(PNI\times NRI\right)}$$

The deceased patient group exhibited significantly higher NCPI values than those in the surviving group (Figure 1E).

Associations of nutritional indicators and clinicopathologic features with OS.

Next, X-Tile software was employed to determine optimal cutoff values for PNI, CONUT, NRI, and NCPI (Supplementary Figure 1), which were determined to be 44.9, 1, 91.3, and 3.7, respectively. Patients were then stratified into High and Low groups based on the cutoff values for each parameter, and clinicopathological characteristics and long-term prognosis (OS) compared between the two groups.

**Figure 2:**
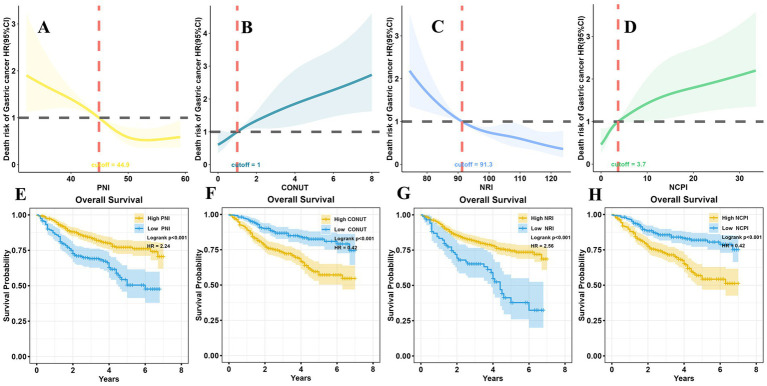
RCS analysis and survival analysis according to nutritional parameters. **(A)** At PNI > 44.9, the risk of death in patients with GC remained relatively stable; however, it significantly increased as PNI decreased to <44.9. **(B)** The risk of death in patients with GC was not significantly changed with CONUT <1; however, it notably increased with CONUT ≥1. **(C)** When NRI > 91.3, the risk of death in GC patients did not vary significantly; conversely, it increased markedly as NRI decreased <91.3. **(D)** The risk of death in patients with GC remained relatively stable with NCPI <3.7; however, it exponentially increased as NCPI rose >3.7. Long-term survival outcomes were notably better in the high PNI group than those in the low PNI group **(E)**, in the low CONUT group than those in the high CONUT group **(F)**, in the high NRI group than those in the low NRI group **(G)**, and in the low NCPI group than those in the high NCPI group **(H)**. RCS, restricted cubic spline; PNI, Prognostic Nutritional Index; CONUT, Controlling Nutritional Status; NRI, Nutritional Risk Index; NCPI, Nutrition-combined Prognostic Index.

Comparisons of the High PNI and High NRI groups with the Low PNI and Low NRI groups, respectively, revealed significant differences: patients in the former groups achieved longer survival times; included higher proportions of survivors and patients with early T and N stage tumors; and more patients with higher BMI, younger age, smaller tumor size, and lower LNR. Conversely, patients in the High PNI and High NRI groups had fewer positive lymph nodes than those in the Low PNI and Low NRI groups, respectively. All differences were significant (Supplementary Table 2). A restricted cubic spline (RCS) diagram indicated a sharp increase in the risk of death from GC as PNI and NRI decreased below their respective cutoff values (Figures 2A,C). Survival analysis further confirmed the superior prognosis of patients in the High PNI and High NRI groups relative to those in the Low PNI (HR = 2.24, *p* < 0.001) and Low NRI (HR = 2.56, *p* < 0.001) groups, respectively (Figures 2E,G).

Further, notable differences were detected in patients in the High CONUT and High NCPI groups compared with those in the Low CONUT and Low NCPI groups, respectively. Those in the former groups had shorter survival times and included higher proportions of patients who died of the disease. Additionally, they included higher percentages of male patients, and of those with advanced age, greater presence of underlying diseases, and higher proportions of advanced T and N stage tumors, as well as higher LNR. Furthermore, individuals in these groups tended to have lower BMI, larger tumor size, and more positive lymph nodes (Supplementary Table 2). RCS illustrated a significant elevation in the risk of death from GC with increasing CONUT and NCPI when they surpassed their respective cutoff values (Figures 2B,D). Survival analysis corroborated these findings, indicating that the prognosis of patients in the High CONUT and High NCPI groups was notably poorer than that of patients in the Low CONUT and Low NCPI groups, respectively (HR = 0.42, *p* < 0.001 for both comparisons; Figures 2F,H).

### 3.3 Regression analysis of nutritional markers

To ascertain whether NCPI represents an independent risk factor for OS in patients with GC, we conducted both univariate and multivariate Cox regression analyses. Our findings revealed that NCPI indeed emerged as an independent risk factor for OS in patients with GC (Supplementary Table 3).

**Figure 3:**
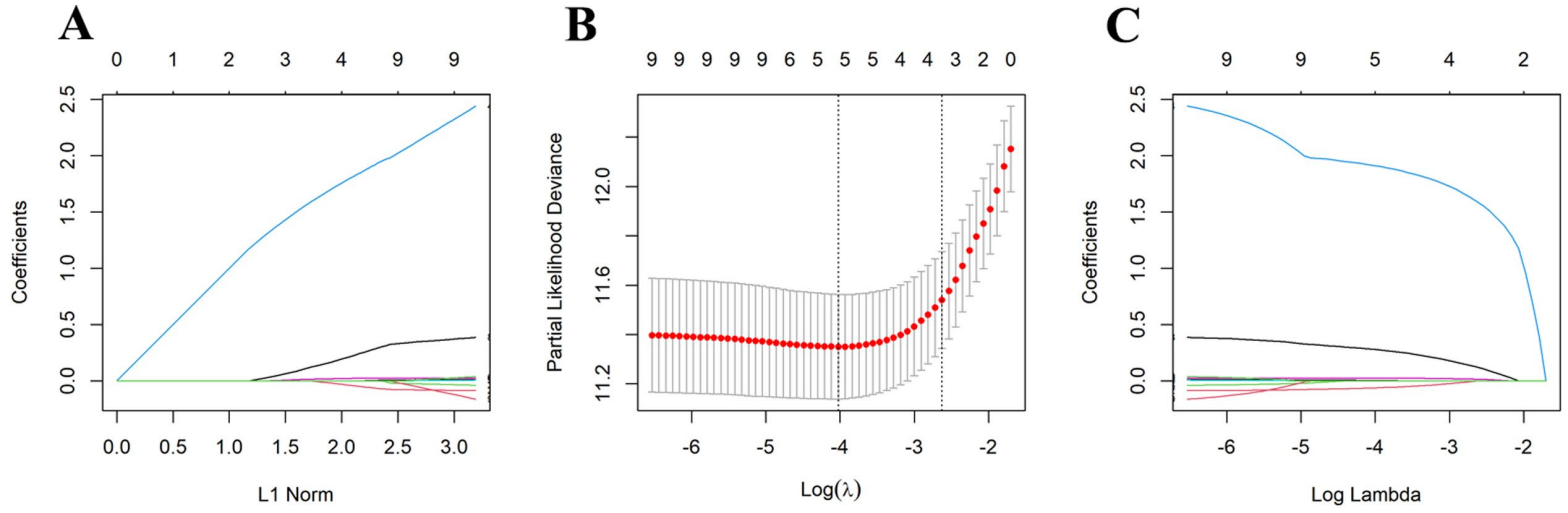
Five clinical and pathological features were selected by LASSO regression analysis at the minimum *λ* value. **(A)** LASSO coefficient path plot, **(B)** LASSO cross-validation curve, **(C)** LASSO feature selection path plot.

To establish a prediction model for OS, we initially divided the data into training and validation sets (ratio, 7:3), with no statistically significant variance between them (Supplementary Table 4). Following univariate Cox regression analysis of the training set, we identified patient age, BMI, tumor size, AJCC T-stage, AJCC N-stage, LNR, positive lymph nodes, chemotherapy, and NCPI as factors associated with OS in patients with GC. Prior to conducting multivariate Cox analysis, these parameters were analyzed by LASSO regression, to mitigate overfitting. Subsequently, based on minimum *λ* (Figure 3), we selected five parameters (age, BMI, AJCC T-stage, LNR, and NCPI) for further multivariate Cox regression analysis. Our results demonstrated that patient BMI (HR: 0.92, 95% CI: 0.86–0.98), AJCC T-stage (HR: 1.42, 95% CI: 1.17–1.72), LNR (HR: 7.57, 95% CI: 3.57–16.08), and NCPI (HR: 1.03, 95% CI: 1.01–1.05) were independent risk factors for OS in patients with GC (Table 3).

**Table 3:** Results of univariate and multivariate Cox regression analyses to identify risk factors associated with overall survival in patients with gastric cancer.

Characteristics	Univariate		*p*	Multivariate		*p*
	HR	95%CI		HR	95%CI	
Age	1.03	1.01–1.05	0.009	1.01	0.99–1.03	0.32
BMI	0.88	0.83–0.94	<0.001	0.92	0.86–0.98	0.02
AJCC T_Stage	1.71	1.44–2.04	<0.001	1.42	1.17–1.72	<0.001
LNR	16.84	8.72–32.51	<0.001	7.57	3.57–16.08	<0.001
NCPI	1.04	1.03–1.06	<0.001	1.03	1.01–1.05	0.004
Gender	1.56	0.99–2.45	0.054			
Histological differentiation	1.06	0.75–1.49	0.74			
Underlying diseases	1.24	0.96–1.6	0.11			
Total_LN	0.98	0.96–1.01	0.18			
Tumor size	1.17	1.06–1.29	0.001			
chemotherapy	3	1.82–4.95	<0.001			
Pathological classification	0.89	0.73–1.09	0.27			
AJCC N_Stage	1.71	1.46–2	<0.001			
Positive_LN	1.07	1.05–1.09	<0.001			
Primary site	0.88	0.67–1.16	0.36			
Complications	0.87	0.72–1.05	0.14			

BMI, Body Mass Index; LN, Lymph Nodes; LNR, Lymph Node Ratio; NCPI, Nutrition-combined Prognostic Index.

### 3.4 Prognostic model establishment and validation

**Figure 4:**
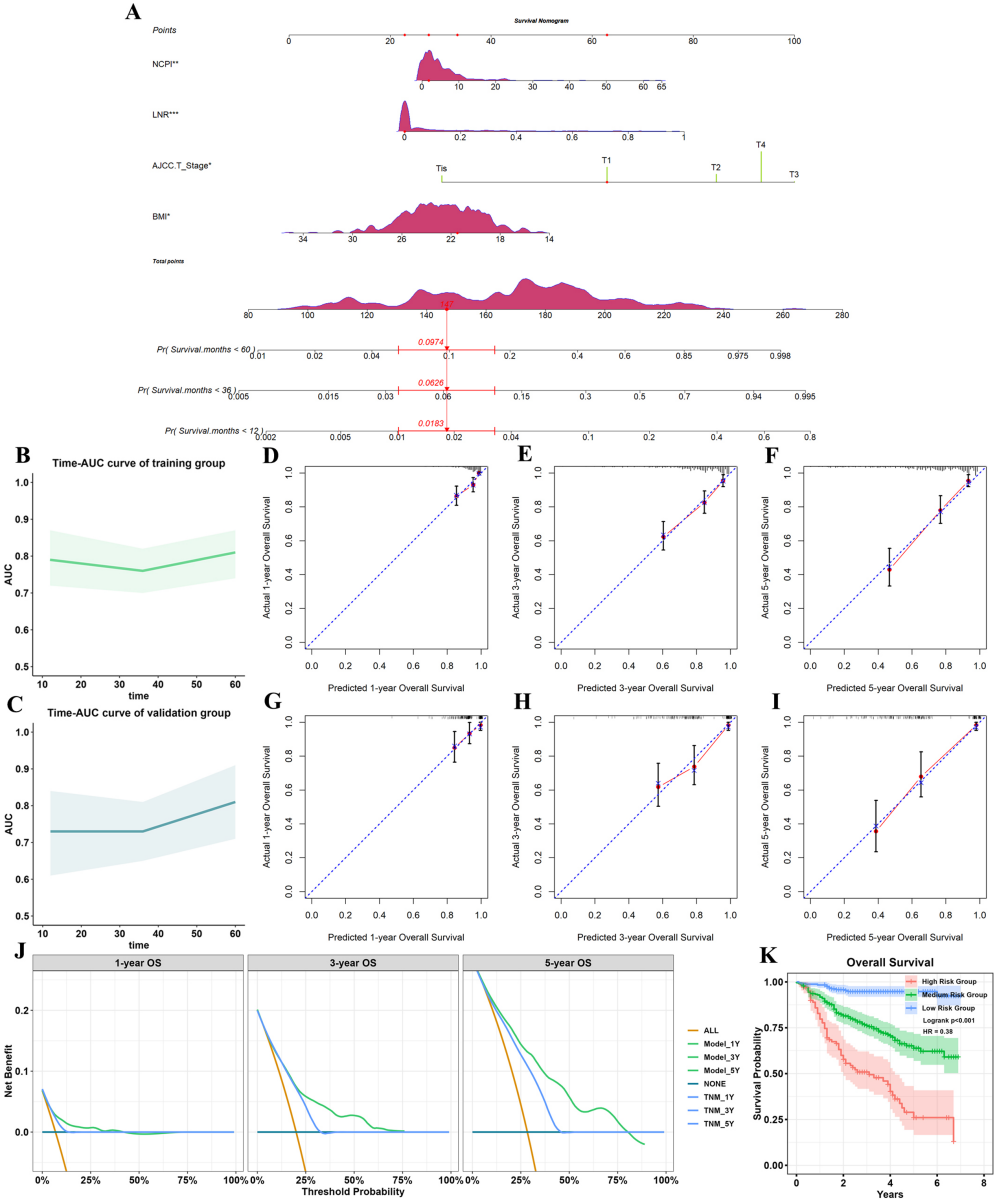
Establishment and validation of a prognostic model. **(A)** Nomogram for predicting 1-, 3-, and 5-year all-cause mortality rates in patients with GC. *p < 0.05; **p < 0.01; ***p < 0.001. Each parameter in the figure has a corresponding point value above it, and point values were summed corresponding to four patient parameters to obtain a total points value. Finally, the predicted probability corresponding to the total point value directly below it was identified. The figure shows an example of a patient with GC who had NCPI = 1.88, LNR = 0, AJCC T-stage T1, and BMI = 21.48, with a total points score of 147, corresponding to 1-, 3-, and 5-year all-cause mortality rates of 0.018, 0.063, and 0.097, respectively; and 1-year, 3-year, and 5-year OS of 0.982, 0.937, and 0.903. Time-AUC curves for the training group **(B)** and the validation group **(C)**. **(D–F)** Calibration curves for 1-, 3-, and 5-year OS for the training group. **(G–I)** Calibration curves for 1-, 3-, and 5-year OS for the validation group. **(J)** Decision analysis curves. When the threshold was >10%, the net gain in 1-and 5-year OS for the predictive model was greater than that for the traditional TNM model; when the threshold is >24%, the 3-year net gain in OS of the prediction model was greater than that of the traditional TNM model. **(K)** Analysis of OS of patients with GC in different risk groups. GC, gastric cancer; NCPI, Nutrition-combined Prognostic Index.

We next generated receiver operating characteristic curves for PNI, CONUT, NRI, and NCPI and calculated AUC values (Supplementary Figure 2; Supplementary Table 5). Despite NCPI exhibiting a higher AUC value than those generated using PNI, CONUT, and NRI, it fell short of expectations as a standalone predictor for GC prognosis. Thus, based on the results of the multivariate Cox analysis, we developed a nomogram for predicting 1-, 3-, and 5-year OS in patients with GC, incorporating four independent risk factors (Figure 4A). Assessment of model differentiation by calculating C-index values revealed favorable performance in both the training (C-index: 0.716, 95% CI: 0.677–0.752) and validation (C-index: 0.77, 95% CI: 0.731–0.806) datasets. Time-AUC curves illustrated stable predictive ability over 1-, 3-, and 5-years OS in both the training and validation datasets (Figures 4B,C). Calibration curves demonstrated close alignment between predicted and actual results (Figures 4D–I). Further, decision curve analysis highlighted the superior clinical utility of our prognostic model over conventional TNM stage (Figure 4J). Patient total scores categorized into high, medium, and low-risk groups using X-Tile software exhibited distinct survival curves (Supplementary Figure 3), with higher scores correlated with inferior prognosis (Figure 4K).

### 3.5 Web-based model calculator

We next created a web-based dynamic nomogram calculator, accessible.^4^ Clinicians can use this tool to promptly predict 1-, 3-, and 5-year OS for a patient by entering the relevant parameters as soon as test results are available in the clinic (Supplementary Figure 4).

## 4 Discussion

GC is a significant and ongoing health concern, ranking fifth and fourth in terms of incidence and mortality rates, respectively (1). The clinical importance of patient nutritional status in managing GC has gained recognition over time; nevertheless, clinicians have struggled to find a straightforward, objective, and accurate method to evaluate this parameter. In this study, we used PNI, CONUT, and NRI as simple, readily available, and objective indicators, to assess individual nutritional status from varying perspectives. Unfortunately, none of these separate indices emerged as independent risk factors for OS. Therefore, we amalgamated the PNI, CONUT, and NRI instruments to devise a novel index, NCPI, with the aim of providing a more comprehensive assessment of individual nutritional status. Our findings reveal that NCPI score correlates with patient prognosis and is an independent risk factor for OS in individuals with GC.

PNI combines data on serum albumin and peripheral lymphocyte counts, which are essential markers for assessing both nutritional and immune status, highlighting the crucial connection between nutrition and immunity. Serum albumin serves as a pivotal indicator of nutritional status, that is closely correlated with degree of malnutrition, and routinely applied in clinical settings to assess and monitor nutritional health. Studies, including those by Oñate-Ocaña et al. (28), have underscored the prognostic significance of serum albumin in the context of GC. Physiological dysregulation of cytokines, such as tumor necrosis factor-alpha and interleukin 6, in patients with tumors may impede albumin synthesis by hepatocytes, influencing cancer progression and neoangiogenesis (29–32). Lymphocytes are pivotal in cellular immune surveillance and inhibition of cancer cell proliferation and migration, as well as having prognostic significance (33–35). Lower peripheral lymphocyte counts are often indicative of inferior patient prognosis (36). Nogueiro et al. reported positive correlations between higher PNI and improved OS and disease-free survival (37). Similarly, studies by Maejima et al. and Dai et al. reported that lower PNI levels were associated with shorter OS and progression-free survival (PFS), as well as inferior clinicopathological parameters (38, 39). Hirahara et al. identified PNI as an independent risk factor for postoperative complications in patients undergoing gastrectomy (40). Consistently, our findings demonstrate that patients in the low PNI group (≤ 44.9) had poorer OS and detrimental clinicopathological features, including advanced tumor stage, larger tumor size, and more positive lymph nodes.

CONUT was initially devised as a comprehensive scoring system to gage both nutritional and immunological status, and has been demonstrated to be correlated with hospitalization duration (41). The CONUT system integrates serum albumin, total cholesterol levels, and lymphocyte counts, encompassing various facets of nutrition to bolster its efficacy in accurately evaluating nutritional health. Research by Okuyama et al. highlighted associations between total cholesterol levels and the progression and prognosis of multiple types of cancer (42). Similarly, de Martino et al. reported a negative correlation between total cholesterol levels and the risk of cancer, including GC (43). One reason for these findings may be that cholesterol, as a major component of cell membranes, is involved in various signaling pathways that mediate tumor development (44). Further, Chen et al. found that patients with elevated CONUT scores experienced poorer PFS and OS (45), while Xiao et al. reported that individuals with high CONUT scores had shorter OS (*p* = 0.005) (15). Additionally, CONUT scores are significantly associated with postoperative complications in patients with GC (*p* < 0.001), particularly anastomotic fistula (*p* = 0.037). The findings of our study are consistent with these observations, in that individuals with high CONUT scores (> 1) had inferior OS and unfavorable clinicopathological characteristics.

NRI, which incorporates patient height, weight, and serum albumin, serves as a comprehensive indicator of nutritional status. A study of patients with esophageal cancer by Cox et al. highlighted the predictive value of baseline NRI < 100 for reduced OS (46). Interestingly, nutritional interventions at baseline improved survival outcomes, whereas interventions at later stages did not yield significant benefits. A retrospective analysis of patients with esophageal squamous cell carcinoma and adenocarcinoma by Clavier et al. corroborated these findings, identifying NRI ≥ 97.5 as an independent prognostic factor (47). Further, research by Oh et al. underscored the significance of NRI in postoperative settings for patients with GC, particularly on the fifth day postoperatively, where lower NRI was associated with increased wound complications (48). In our study, we observed an association between NRI ≤ 91.3 and unfavorable patient clinicopathological characteristics, with lower NRI values indicating a higher risk of patient mortality, suggesting inferior prognosis.

Although we found that PNI, CONUT, and NRI were closely associated with clinicopathologic characteristics and OS in patients with GC, none of these indicators emerged as independent prognostic factors for OS following multivariate analysis. Therefore, we devised the novel nutritional parameter, NCPI, which integrates PNI, CONUT, and NRI. Through univariate, LASSO, and multivariate analyses, we identified NCPI, BMI, AJCC T-stage, and LNR as independent risk factors for OS following radical surgery for GC. NCPI >3.7 was indicative of detrimental clinicopathologic characteristics and inferior OS, where the risk of patient mortality escalated exponentially with increasing NCPI. Based on the results of multivariate analysis, we established a nomogram for predicting OS. C-index values of our model in the training and validation sets were 0.716 and 0.77, respectively, which surpassed the performance of the traditional TNM system. Additionally, calibration curves demonstrated close alignment between predicted and actual values. Furthermore, decision analysis curves indicated a greater net benefit of our predictive model relative to application of the traditional TNM system.

While our study provides valuable insights, it is important to acknowledge its limitations and exercise caution in interpreting the NCPI and nomogram. First, the retrospective nature of our study may have introduced selective bias. Second, our sample size was relatively small, and the follow-up period was relatively short, which could limit the generalizability of our findings. Third, as neoadjuvant therapy may affect some nutritional parameters, we did not include patients undergoing this type of treatment in our analysis. Fourth, while internal data validation was performed, external data validation is currently pending. Future endeavors should involve a multicenter prospective study, encompassing a sufficiently large sample size, with diverse population representation and an extended follow-up duration for validation purposes.

## 5 Conclusion

The findings of this study underscore the significant link between nutritional status and OS in patients with GC. While we found that PNI, CONUT, and NRI were all associated with clinicopathologic features and OS in the context of GC, none were independent prognostic factors for OS. Therefore, we amalgamated PNI, CONUT, and NRI into a novel nutritional parameter, NCPI. Our data demonstrate that NCPI, BMI, AJCC T-stage, and LNR, are independent factors associated with OS following radical GC surgery. Leveraging these findings, we developed a nomogram and a web calculator to predict OS in patients with GC, which demonstrated superior predictive power and net benefit relative to traditional TNM stage-based predictions. Moving forward, it will be imperative to dynamically monitor patient nutritional status, implement interventions for those at nutritional risk, and further explore the nuanced relationship between nutritional risk and prognosis in the context of GC.

## Data Availability

The raw data supporting the conclusions of this article will be made available by the authors, without undue reservation.

## Footnotes

^1^ https://gco.iarc.fr/

^2^ https://medicine.yale.edu/lab/rimm/research/software/

^3^ https://www.r-project.org/

^4^ https://jasonidea.shinyapps.io/gc_ncpi/
