# Supplementary material for: A novel integrated nutrition-combined prognostic index for predicting overall survival after radical gastrectomy
Source: Front Nutr. 2024 Nov 18;11:1438319. doi: 10.3389/fnut.2024.1438319 (PMC11608950; doi:10.3389/fnut.2024.1438319)
Supplement: Supplementary file 1 [file Data_Sheet_1.docx]

# Supplementary Figure Legends


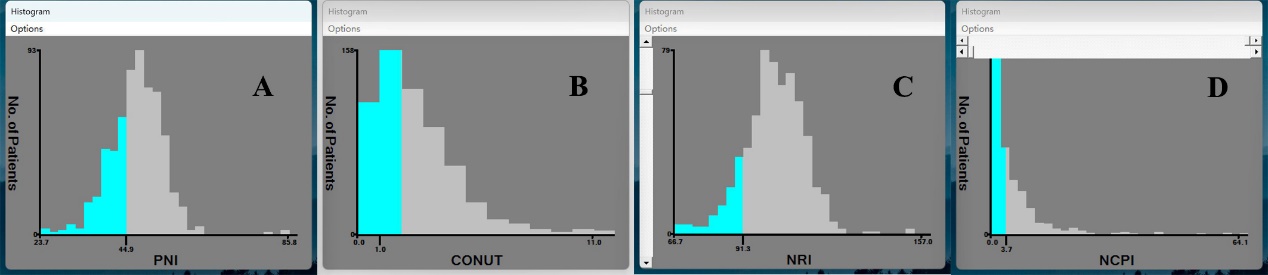


Supplementary Figure 1. Optimal cutoff values for nutrient parameters. A. The optimal cutoff value for PNI is 44.9. B. The optimal cutoff value for CONUT is 1. C. The optimal cutoff value for NRI is 91.3. D. The optimal cutoff value for NCPI is 3.7.


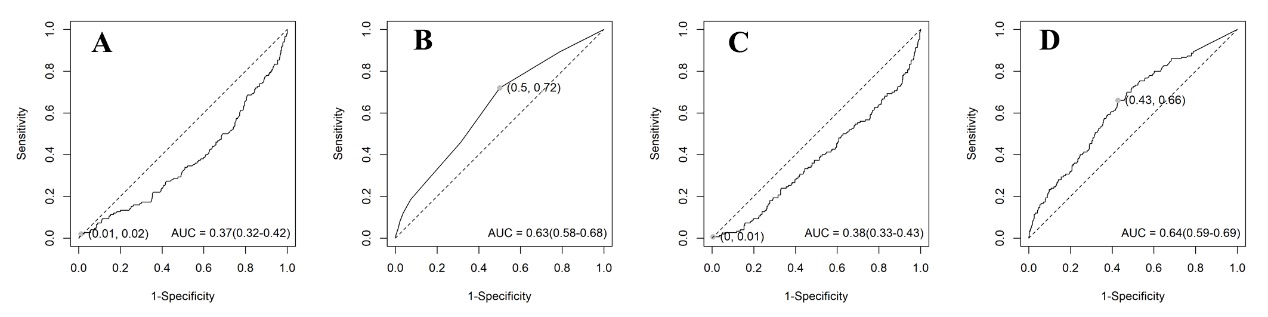


Supplementary Figure 2. ROC curves for nutrient parameters. A. ROC curve for PNI. B. ROC curve for CONUT. C. ROC curve for NRI. D. ROC curve for NCPI.


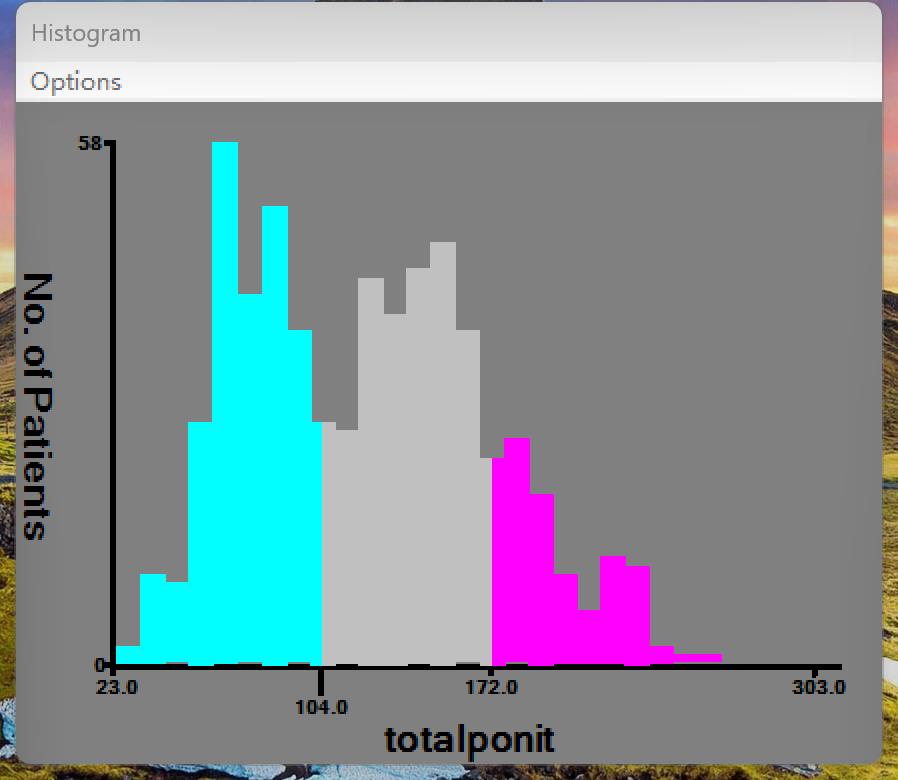


Supplementary Figure 3. All patients were categorized into high, medium, and low risk groups.


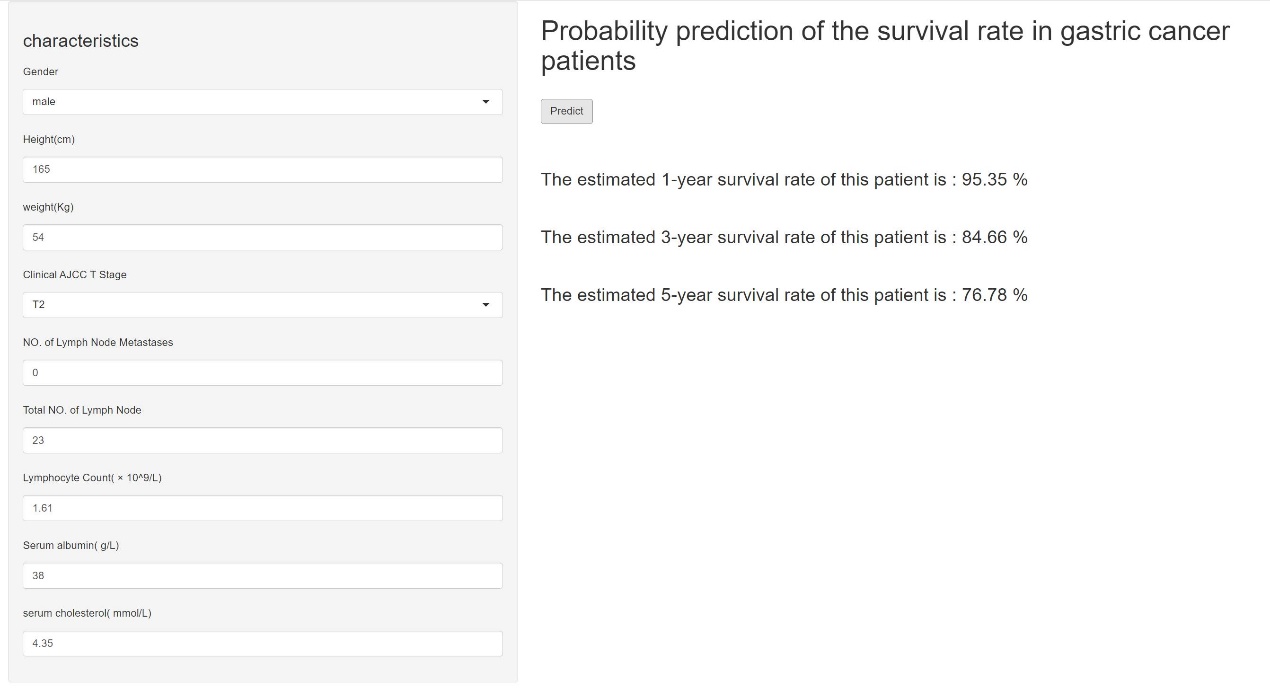


Supplementary Figure 4. Dynamic network calculator. For example, a male gastric cancer patient with a height of 165 cm, a weight of 54 Kg, an AJCC T-stage of T2, a positive lymph node number of 0, a total lymph node count of 23, a lymphocyte count of 1.61 × 10^9/L, an albumin of 38 g/L, and a cholesterol of 4.35 mmol/L. After inputting the above parameters into the calculator, and then clicking on [Predict], the patient can be predicted that the 1-year survival rate is roughly 95.35%, the 3-year survival rate is roughly 84.66%, and the 5-year survival rate drops to 76.78%.

# Supplementary Tables

| Supplementary TABLE 1: Univariate and multivariate Cox regression analyses(excluding NCPI) | | | | | | |
| --- | --- | --- | --- | --- | --- | --- |
| Characteristics | Univariate | | P | Multivariate | | p |
|  | HR | 95%CI |  | HR | 95%CI |  |
| Gender | 1.47 | 1.01-2.13 | 0.04 | 1.25 | 0.85-1.83 | 0.26 |
| Age | 1.02 | 1.01-1.04 | 0.004 | 1.01 | 1-1.03 | 0.13 |
| BMI | 0.9 | 0.85-0.95 | <0.001 | 0.88 | 0.79-0.99 | 0.03 |
| Tumor size | 1.21 | 1.13-1.3 | <0.001 | 1.03 | 0.94-1.13 | 0.54 |
| Pathological classification | 0.79 | 0.65-0.96 | 0.018 | 0.89 | 0.71-1.1 | 0.28 |
| AJCC T_Stage | 1.78 | 1.53-2.06 | <0.001 | 1.47 | 1.21-1.79 | <0.001 |
| AJCC N_Stage | 1.7 | 1.49-1.94 | <0.001 | 0.87 | 0.67-1.13 | 0.31 |
| LNR | 16.48 | 9.47-28.69 | <0.001 | 12.22 | 3.52-42.46 | <0.001 |
| Positive_LN | 1.07 | 1.06-1.09 | <0.001 | 1 | 0.97-1.04 | 0.88 |
| chemotherapy | 2.98 | 1.97-4.49 | <0.001 | 0.99 | 0.61-1.62 | 0.98 |
| PNI | 0.95 | 0.92-0.97 | <0.001 | 0.97 | 0.9-1.04 | 0.38 |
| COUNT | 1.18 | 1.11-1.26 | <0.001 | 1.1 | 0.97-1.24 | 0.12 |
| NRI | 0.97 | 0.95-0.98 | <0.001 | 1.03 | 0.98-1.09 | 0.24 |
| Primary site | 0.83 | 0.67-1.04 | 0.1 |  |  |  |
| Histological differentiation | 1.02 | 0.77-1.35 | 0.9 |  |  |  |
| Underlying diseases | 1.21 | 0.98-1.51 | 0.08 |  |  |  |
| Total_LN | 0.99 | 0.97-1.01 | 0.26 |  |  |  |
| Complications | 0.87 | 0.74-1.02 | 0.08 |  |  |  |
| BMI, Body Mass Index; LN, Lymph Nodes; LNR, Lymph Node Ratio;  NCPI, Nutrition-combined Prognostic Index | | | | | | |

| Supplementary TABLE 3: Univariate and multivariate Cox regression analyses (including NCPI) | | | | | | |
| --- | --- | --- | --- | --- | --- | --- |
| Characteristics | Univariate | | P | Multivariate | | p |
|  | HR | 95%CI |  | HR | 95%CI |  |
| Gender | 1.47 | 1.01-2.13 | 0.04 | 1.23 | 0.84-1.79 | 0.29 |
| Age | 1.02 | 1.01-1.04 | 0.004 | 1.01 | 1-1.03 | 0.14 |
| BMI | 0.9 | 0.85-0.95 | <0.001 | 0.94 | 0.88-0.99 | 0.02 |
| Tumor size | 1.21 | 1.13-1.3 | <0.001 | 1.03 | 0.94-1.12 | 0.59 |
| Pathological classification | 0.79 | 0.65-0.96 | 0.02 | 0.89 | 0.71-1.1 | 0.29 |
| AJCC T_Stage | 1.78 | 1.53-2.06 | <0.001 | 1.49 | 1.22-1.81 | <0.001 |
| AJCC N_Stage | 1.7 | 1.49-1.94 | <0.001 | 0.85 | 0.66-1.11 | 0.23 |
| LNR | 16.48 | 9.47-28.69 | <0.001 | 12.23 | 3.56-42.06 | <0.001 |
| Positive_LN | 1.07 | 1.06-1.09 | <0.001 | 1.01 | 0.97-1.04 | 0.71 |
| chemotherapy | 2.98 | 1.97-4.49 | <0.001 | 1 | 0.61-1.62 | 0.99 |
| NCPI | 1.03 | 1.02-1.05 | <0.001 | 1.02 | 1-1.03 | 0.02 |
| Primary site | 0.83 | 0.67-1.04 | 0.11 |  |  |  |
| Histological differentiation | 1.02 | 0.77-1.35 | 0.9 |  |  |  |
| Underlying diseases | 1.21 | 0.98-1.51 | 0.08 |  |  |  |
| Total_LN | 0.99 | 0.97-1.01 | 0.26 |  |  |  |
| Complications | 0.87 | 0.74-1.02 | 0.08 |  |  |  |
| BMI, Body Mass Index; LN, Lymph Nodes; LNR, Lymph Node Ratio;  NCPI, Nutrition-combined Prognostic Index | | | | | | |

| Supplementary TABLE 4: Baseline features after grouping | | | | |
| --- | --- | --- | --- | --- |
| Characteristic | Overall N = 609 | Training cohort N = 427 | Validation cohort N = 182 | p-value |
| Vital status |  |  |  | 0.3 |
| death | 150 (25%) | 100 (23%) | 50 (27%) |  |
| survival | 459 (75%) | 327 (77%) | 132 (73%) |  |
| Gender |  |  |  | 0.3 |
| female | 194 (32%) | 141 (33%) | 53 (29%) |  |
| male | 415 (68%) | 286 (67%) | 129 (71%) |  |
| Age | 66 (59, 72) | 67 (59, 73) | 66 (59, 71) | 0.6 |
| Underlying diseases |  |  |  | >0.9 |
| 0 | 424 (70%) | 293 (69%) | 131 (72%) |  |
| 1 | 137 (22%) | 98 (23%) | 39 (21%) |  |
| 2 | 43 (7.1%) | 32 (7.5%) | 11 (6.0%) |  |
| 3 | 4 (0.7%) | 3 (0.7%) | 1 (0.5%) |  |
| 4 | 1 (0.2%) | 1 (0.2%) | 0 (0%) |  |
| BMI | 22.76 (20.57, 24.61) | 22.76 (20.56, 24.74) | 22.73 (20.76, 24.13) | 0.5 |
| Tumor size | 3.00 (1.80, 4.00) | 2.50 (1.80, 4.00) | 3.00 (1.85, 4.00) | 0.2 |
| Histological differentiation |  |  |  | 0.2 |
| Well differentiated | 9 (1.5%) | 8 (1.9%) | 1 (0.5%) |  |
| Moderately differentiated | 139 (23%) | 89 (21%) | 50 (27%) |  |
| Poorly differentiated | 419 (69%) | 300 (70%) | 119 (65%) |  |
| Unclassified | 42 (6.9%) | 30 (7.0%) | 12 (6.6%) |  |
| Pathological classification |  |  |  | 0.4 |
| adenocarcinoma | 497 (82%) | 351 (82%) | 146 (80%) |  |
| mucinous carcinoma | 10 (1.6%) | 5 (1.2%) | 5 (2.7%) |  |
| signet-ring cell carcinoma | 70 (11%) | 49 (11%) | 21 (12%) |  |
| Adenosquamous carcinoma | 1 (0.2%) | 1 (0.2%) | 0 (0%) |  |
| squamous cell carcinoma | 5 (0.8%) | 5 (1.2%) | 0 (0%) |  |
| carcinoma | 26 (4.3%) | 16 (3.7%) | 10 (5.5%) |  |
| AJCC T_Stage |  |  |  | >0.9 |
| Tis | 65 (11%) | 46 (11%) | 19 (10%) |  |
| T1 | 150 (25%) | 106 (25%) | 44 (24%) |  |
| T2 | 79 (13%) | 55 (13%) | 24 (13%) |  |
| T3 | 7 (1.1%) | 4 (0.9%) | 3 (1.6%) |  |
| T4 | 308 (51%) | 216 (51%) | 92 (51%) |  |
| AJCC N_Stage |  |  |  | 0.8 |
| N0 | 326 (54%) | 233 (55%) | 93 (51%) |  |
| N1 | 95 (16%) | 67 (16%) | 28 (15%) |  |
| N2 | 79 (13%) | 53 (12%) | 26 (14%) |  |
| N3 | 109 (18%) | 74 (17%) | 35 (19%) |  |
| LNR | 0.00 (0.00, 0.19) | 0.00 (0.00, 0.18) | 0.00 (0.00, 0.21) | 0.5 |
| Positive_LN | 0.0 (0.0, 4.0) | 0.0 (0.0, 4.0) | 0.0 (0.0, 4.0) | 0.5 |
| Total_LN | 22 (17, 28) | 22 (17, 28) | 21 (16, 28) | 0.2 |
| Primary site |  |  |  | 0.5 |
| upper third | 121 (20%) | 83 (19%) | 38 (21%) |  |
| middle third | 281 (46%) | 204 (48%) | 77 (42%) |  |
| lower third | 207 (34%) | 140 (33%) | 67 (37%) |  |
| chemotherapy | 370 (61%) | 258 (60%) | 112 (62%) | 0.8 |
| Complications |  |  |  | 0.2 |
| fistula | 47 (7.7%) | 39 (9.1%) | 8 (4.4%) |  |
| infection | 29 (4.8%) | 22 (5.2%) | 7 (3.8%) |  |
| other | 26 (4.3%) | 17 (4.0%) | 9 (4.9%) |  |
| none | 507 (83%) | 349 (82%) | 158 (87%) |  |
| BMI, Body Mass Index; LN, Lymph Nodes; LNR, Lymph Node Ratio | | | | |

| Supplementary TABLE 5: AUC of nutritional parameters | | |
| --- | --- | --- |
| Parameters | AUC | 95%CI |
| PNI | 0.369 | 0.317-0.422 |
| CONUT | 0.628 | 0.578-0.678 |
| NRI | 0.380 | 0.327-0.432 |
| NCPI | 0.636 | 0.585-0.687 |
| PNI, Prognostic Nutritional Index;  CONUT, Controlling Nutritional Status;  NRI, Nutritional Risk Index;  NCPI, Nutrition-combined Prognostic Index. | | |
